# Analysis of the *Bacillus cereus* SpoIIS antitoxin-toxin system reveals its three-component nature

**DOI:** 10.3389/fmicb.2015.00808

**Published:** 2015-08-06

**Authors:** Jana Melničáková, Zuzana Bečárová, Jana Makroczyová, Imrich Barák

**Affiliations:** Institute of Molecular Biology, Slovak Academy of SciencesBratislava, Slovakia

**Keywords:** *Bacillus cereus*, toxin-antitoxin system, SpoIIS, programmed cell death, *Bacillus subtilis*

## Abstract

Programmed cell death in bacteria is generally associated with two-component toxin-antitoxin systems. The SpoIIS toxin-antitoxin system, consisting of a membrane-bound SpoIISA toxin and a small, cytosolic antitoxin SpoIISB, was originally identified in *Bacillus subtilis*. In this work we describe the *Bacillus cereus* SpoIIS system which is a three-component system, harboring an additional gene *spoIISC*. Its protein product serves as an antitoxin, and similarly as SpoIISB, is able to bind SpoIISA and abolish its toxic effect. Our results indicate that SpoIISC seems to be present not only in *B. cereus* but also in other Bacilli containing a SpoIIS toxin-antitoxin system. In addition, we show that *B. cereus* SpoIISA can form higher oligomers and we discuss the possible role of this multimerization for the protein's toxic function.

## Introduction

Programmed cell death (PCD) is a genetically regulated system in which a bacterial cell is able to commit suicide in response to a variety of different stresses. This response includes cell lysis or growth inhibition induced by harsh environmental conditions such as starvation or antibiotic treatment, active mother cell lysis during sporulation to release the spore, or altruistic suicide to release cell content to provide the nutrients required for the normal development of the remaining bacterial population (Engelberg-Kulka et al., 2006). PCD is usually mediated by a pair of toxin/antitoxin (TA) genes. Toxins are always highly stabile proteins. Their antidotes, the antitoxins, are usually labile proteins or small RNAs. TA systems are classified according to the nature of the antitoxin. Type I and III are small RNAs which either inhibit the synthesis of the toxin or capture it. Examples include the type I *hok-sok* system (Pedersen and Gerdes, 1999) and the type III ToxIN system (Fineran et al., 2009). Types II, IV, and V, on the other hand, are all proteins. They include the type II *mazEF* TA system (Gerdes et al., 2005), the type IV *yeeU-yeeV* system (Masuda et al., 2012), and the type V *ghoT-ghoS* system (Wang et al., 2012). These three types are distinguished based on their mode of action. The type II antitoxin is a small protein with an N-terminal DNA-binding domain and a C-terminal toxin-bonding domain, the type IV antitoxin is an antagonist of its cognate toxin and competes with it in binding to its target, and the type V antitoxin is an endoribonuclease that degrades the toxin-encoding mRNA (Goeders and Van Melderen, 2014).

Many bacteria harbor genes for TA systems on plasmids (Ruiz-Echevarría et al., 1995; Gerdes et al., 1997; Sayeed et al., 2000; Van Melderen, 2001; Camacho et al., 2002). These genes are part of a mechanism called post-segregational killing, which ensures that their host plasmids are retained in the daughter cells of a growing bacterial population. In this process, the stable, long-lived toxin kills those daughter cells which do not inherit the plasmid encoding the labile antitoxin (Gerdes et al., 1986; Lehnherr and Yarmolinsky, 1995; Hayes, 2003). Other bacterial species contain numerous toxin-antitoxin genes on their chromosome (Hayes, 2003; Tsilibaris et al., 2007; Van Melderen and Saavedra De Bast, 2009). Chromosomal TA systems may serve to prevent the spread of mobile genetic elements such as phages or plasmids; they are typically involved in the general stress response and in guarding against DNA loss (reviewed in Schuster and Bertram, 2013).

The *spoIIS* locus was originally identified on the *Bacillus subtilis* chromosome during a study of the genetic mutants that block sporulation after the formation of the polar septum (Adler et al., 2001). Formerly, the locus was thought to consist of two genes, *spoIISA* coding for the toxin and *spoIISB* for proteic antitoxin (Adler et al., 2001), thus classifying as type II TA system. A condition-dependent analysis of the transcription of all *B. subtilis* genes indicated that a third transcriptionally active region, S458, might be present in the *spoIIS* operon (Nicolas et al., 2012), which we name *spoIISC*. Inactivation of the *spoIISA* toxin gene has no effect on sporulation, but inactivation of the *spoIISB* antitoxin gene decreases sporulation efficiency by four orders of magnitude. Furthermore, disruption of *spoIISA* in a *spoIISB* null mutant restores sporulation. Thus, SpoIISB is required for sporulation only if SpoIISA is present in the cell (Adler et al., 2001). The morphological consequence of an artificially induced higher level of toxin expression is the formation of plasmolysis zones in the cytoplasmic membrane, leading to the death of the cell. The transcription of *spoIISA, spoIISB*, and *spoIISC* is upregulated during sporulation from four to up to eight hours (Nicolas et al., 2012); however, the expression of SpoIISA is independent of the crucial sporulation initiation transcription factor, Spo0A (Rešetárová et al., 2010). Production of the SpoIISA toxin is also induced during ethanol stress and nutrient deprivation. During starvation, the production of SpoIISB was detected, which suggests that SpoIISB is able to diminish the toxic effect of SpoIISA. Moreover, SpoIISB is also produced during swarming and at times of high cell density. There is presently only a little information about *spoIISC*, but it is known that its transcription is activated during both sporulation and biofilm formation (Nicolas et al., 2012). The SpoIISA toxin is neutralized by the formation of a tight complex with the SpoIISB antitoxin. The crystal structure of this complex revealed that SpoIISB and the cytoplasmic domain of SpoIISA form a heterotetrameric complex with C-SpoIISA_2_:SpoIISB_2_ stoichiometry (Florek et al., 2011).

Homologs of SpoIISA and SpoIISB proteins have also been identified among other Bacillus species, but they display only a low level of homology. Both *B. subtilis* and *B. cereus* SpoIISA inhibit the growth of *E. coli* cells, and the SpoIISB antitoxin is able to neutralize SpoIISA toxicity in *E. coli* (Florek et al., 2008).

In the present study we analyze the *spoIIS* operon in *B. cereus* ATCC 14579. Even though a third trancriptionally active region in the *spoIIS* operon of *B. subtilis* was identified, it is unclear whether its product is really part of this TA system. We have found that both *B. subtilis* and *B. cereus spoIISC* encode an antitoxin that is able to diminish SpoIISA toxicity in *E. coli*. As in *B. subtilis*, the *B. cereus spoIIS* operon consists of three genes: *spoIISA, spoIISB*, and *spoIISC*. Using a bacterial two hybrid system we show that *B. cereus* C-SpoIISA interacts with other C-SpoIISA molecules, as well as with SpoIISB and SpoIISC. These new positive interactions, identified *in vivo*, were also confirmed *in vitro* using a pull-down assay. *In vitro* analysis of the oligomeric states of *B. cereus* C-SpoIISA revealed that the soluble C-SpoIISA exists in monomeric, dimeric and trimeric forms.

## Materials and methods

### Bacterial strains, growth conditions, and media

The bacterial strains *E. coli* XL1-BLUE, DH5α, and MM294 were used for routine DNA manipulations. The *E. coli* BTH101 reporter strain was employed in the bacterial adenylate cyclase-based two-hybrid system. *E. coli* BL21 (λDE3) cells were employed in expression of recombinant protein. *E. coli* cells were grown at 37°C, 28°C or room temperature in LB (Ausubel et al., 1987) or SOC medium (Hanahan, 1983) or on agar plates. When required, the medium was supplemented with appropriate antibiotics and other additives. *E. coli* transformation and DNA manipulations were performed using standard protocols (Sambrook et al., 1989).

### The kill/rescue assay cultivation

To evaluate the effect of the expression of *B. cereus spoIIS* genes on the growth of *E. coli* MM294, a single colony of bacterial cells was resuspended in 100 μl of LB and grown overnight on LB agar plates. The bacterial lawn was washed off with 1 ml LB and this primary culture was used to inoculate a second cell generation in LB containing 100 μg ml^−1^ ampicillin and 0.5% glucose (w/v). The starting optical density (OD_600_) of the cell cultures was 0.05–0.06. The cells were cultivated at 37°C in an orbital shaker at 150 rpm and growth was monitored by measuring the OD_600_ in 1-h intervals. When the OD_600_ reached 0.4, *spoIIS* expression was induced by the addition of l-arabinose to a final concentration of 0.02% (w/v).

### Recombinant plasmid construction

All bacterial strains and plasmids used in this study are listed in Table 1. All primers for cloning were designed for the PCR amplification of specific genes and regulatory regions and are listed in Table 2. Chromosomal DNA of *B. subtilis* PY79 (Youngman et al., 1984) and *Bacillus cereus* ATCC 14579 was used for amplification of *spoIIS* genes.

**Table 1:** **Strains and plasmids used in this study**.

**Strain**	**Genotype or description**	**Reference or origin**
***E. coli***		
MM294	*F*^−^ *endA1 hsdR17 (rk*^−^*, mk) supE44 thi-1 recA*^+^	Meselson and Yuan, 1968
BTH101	*F*^−^ *cya-99 araD139 galE15 galK16 rpsL1(Str*^R^*) hsdR2 mcrA1 mcrB1*	Karimova et al., 1998
DH5α	*F' Iq supE44* Δ*lacU169 (*φ*80 lacZ*Δ*M15) hsdR17 recA1 endA1 gyrA96 thi-1 relA1*	Meselson and Yuan, 1968
XL1-BLUE	Δ*(mcrA)183 (mcrCB-hsdSMR-mrr)173 endA1 supE44 thi-1 recA*^1^ *gyrA96 relA lac (F' proAB lacI*^q^ Δ*M15Tn5 kan')*	Stratagene
IB890	pBAD24 in MM294	Florek et al., 2008
IB926	pBAD24-BCIISA in MM294	Florek et al., 2008
**PLASMIDS USED IN KILL/RESCUE ASSAY**		
pBAD24	Amp^R^ *araC;* P_BAD_ promoter	Guzman et al., 1995
pBADCIISA	Amp^R^ *araC*; P_BAD_ promoter, *B. cereus spoIISA*-like gene	Florek et al., 2008
pBADIISAB Bc	Amp^R^ *araC*; P_BAD_ promoter, *B. cereus spoIISAB*-like genes	This study
pBADIISC Bc	Amp^R^ *araC*; P_BAD_ promoter, *B. cereus spoIISC*-like gene	This study
pBADIISAC Bc	Amp^R^ *araC*; P_BAD_ promoter, *B. cereus spoIISAC*-like genes	This study
pBADIISA Bs	Amp^R^ *araC*; P_BAD_ promoter, *B. subtilis spoIISA*	This study
pBADIISAB Bs	Amp^R^ *araC*; P_BAD_ promoter, *B. subtilis spoIISAB*	This study
pBADIISC Bs	Amp^R^ *araC*; P_BAD_ promoter, *B. subtilis spoIISC*-like gene	This study
pBADIISAC Bs	Amp^R^ *araC*; P_BAD_ promoter, *B. subtilis spoIISAC*	This study
**PLASMIDS FOR TESTING PROTEIN-PROTEIN INTERACTIONS** ***IN VITRO***		
pET15b	Amp^R^; *T7lac* promoter	Novagen
pETDuet-1	Amp^R^; *T7lac* promoter	Novagen
pET15b-Bc-CIISA	Amp^R^; *T7lac* promoter, *B. cereus C-spoIISA*	Laboratory stock
pET15b-Bc-HCIISA	Amp^R^; *T7lac* promoter, His_6_ tag fused with *B. cereus C-spoIISA*	This study
pETDuet-Bc-IISC	AmpR; *T7lac* promoter, *B. cereus spoIISC*	This study
pETDuet-Bc-HCIISAC	Amp^R^; *T7lac* promoter, His_6_ tag fused with *B. cereus C-spoIISA, T7lac* promoter, *B. cereus spoIISC*	This study
pETDuetCIISA Bc	Amp^R^; *T7lac* promoter, His_6_ tag fused with *B. cereus C-spoIISA*	This study
pETDuetIISB Bc	Amp^R^; *T7lac* promoter, *B. cereus spoIISB* fused with S-tag	This study
pETDuetCIISAB Bc	Amp^R^; *T7lac* promoter His_6_-tag fused with *B. cereus C-spoIISA, T7lac* promoter, *B. cereus spoIISB* fused with S-tag	This study
**PLASMIDS FOR THE BACTERIAL TWO-HYBRID SYSTEM**		
pKT25	Kan^R^; *P*_*lac*_ promoter, *T25*	Karimova et al., 1998
pKNT25	Kan^R^; *P*_*lac*_ promoter, *T25*	Karimova et al., 1998
pUT18	Amp^R^; *P*_*lac*_ promoter, *T18*	Karimova et al., 1998
pUT18C	Amp^R^; *P*_*lac*_ promoter, *T18*	Karimova et al., 1998
pKT25-zip	Kan^R^; *P*_*lac*_ promoter, *T25* fused with *zip*	Karimova et al., 1998
pUT18C-zip	Amp^R^; *P*_*lac*_ promoter, *T18* fused with *zip*	Karimova et al., 1998
pKTCIISA Bc	Kan^R^; *P*_*lac*_ promoter, *T25* fused with *B. cereus C-SpoIISA*	This study
pKNTCIISA Bc	Kan^R^; *P*_*lac*_ promoter, *B. cereus C-spoIISA* fused with *T25*	This study
pUTCIISA Bc	Amp^R^; *P*_*lac*_ promoter, *B. cereus C-spoIISA* fused with *T18*	This study
pUTCCIISA Bc	Amp^R^; *P*_*lac*_ promoter, *T18* fused with *B. cereus C-spoIISA*	This study
pUTIISB Bc	Amp^R^; *P*_*lac*_ promoter, *B. cereus spoIISB* fused with *T18*	This study
pUTCIISB Bc	Amp^R^; *P*_*lac*_ promoter, *T18* fused with *B. cereus spoIISB*	This study
pUTIISC Bc	Amp^R^; *P*_*lac*_ promoter, *B. cereus spoIISC* fused with *T18*	This study
pUTCIISC Bc	Amp^R^; *P*_*lac*_ promoter, *T18* fused with *B. cereus spoIISC*	This study
pKTIISC Bc	Kan^R^; *P*_*lac*_ promoter, *T25* fused with *B. cereus spoIISC*	This study
pKNTIISC Bc	Kan^R^; *P*_*lac*_ promoter, *B. cereus spoIISC* fused with *T25*	This study
pKTCIISA Bs	Kan^R^; *P*_*lac*_ promoter, *T25* fused with *B. subtilis C-spoIISA*	This study
pKNTCIISA Bs	Kan^R^; *P*_*lac*_ promoter, *B. subtilis C-spoIISA* fused with *T25*	This study
pUTCIISA Bs	Amp^R^; *P*_*lac*_ promoter, *B. subtilis C-spoIISA* fused with *T18*	This study
pUTCCIISA Bs	Amp^R^; *P*_*lac*_ promoter, *T18* fused with *B. subtilis C-spoIISA*	This study
pUTIISB Bs	Amp^R^; *P*_*lac*_ promoter, *B. subtilis spoIISB* fused with *T18*	This study
pUTCIISB Bs	Amp^R^; *P*_*lac*_ promoter, *T18* fused with *B. subtilis spoIISB*	This study
pUTIISC Bs	Amp^R^; *P*_*lac*_ promoter, *B. subtilis spoIISC* fused with *T18*	This study
pUTCIISC Bs	Amp^R^; *P*_*lac*_ promoter, *T18* fused with *B. subtilis spoIISC*	This study
pKTIISC Bs	Kan^R^; *P*_*lac*_ promoter, *T25* fused with *B. subtilis spoIISC*	This study
pKNTIISC Bs	Kan^R^; *P*_*lac*_ promoter, *B. subtilis spoIISC* fused with *T25*	This study

**Table 2:** **Primers used in this study**.

**Primer**	**Sequence (5′–3′), restriction sites are in bold**	**Final construct**
SP/Bc-CIISA/XhoI	TCATCATCA**CTCGAG**GAAATATGGGGTGCGAAATT	pET15b-Bc-HCIISA
ASP/Bc-CIISABamE	TCATCATCA**GGATCC**TTTACTAAAATAACTATGAT	
SP/BcIISB/NdeI	TCATCATCA**CATATG**GTGATTGTAGTGGTAAAAGA	pETDuet-Bc-IISC
ASP/BcIISB/XhoI	TCATCATCA**CTCGAG**TACACTTATGATTTTCTTTT	
SP/IISA/NcoI	TCATCATCA**CCATGG**ATGATCTCTAACATTCGAAT	pBADIISAB Bc
ASP/IISB/HindIII	TCATCATCA**AAGCTT**GCAAATGTAGAAAGAGTGTA	
SP/IISCBc/PstI	TCATCATCA**CTGCAG**TGAAAAGGGGGAGAAGAGATG	pBADIISC Bc
ASP/IISCBc/HindIII	TCATCATCA**AAGCTT**ATGCTCTATGCATTTTCTTT	
SP/IISABc/EcoRI	TCATCATCA**GAATTC**ATGATCTCTAACATTCGAAT	pBADIISAC (via pBADIISC Bc)
ASP/IISABc/NcoI	TCATCATCA**CCATGG**TAGAAGAAAAGGACAGAAAA	
SP/CIISABc/BamHI	TCATCATCA**GGATCC**CGAAATATGGGGTGCGAAATT	All four BACTH vectors carrying CIISA Bc
ASP/CIISABcSTOP/EcoRI	TCATCATCA**GAATTC**GATTCTGTCCTTATTTACTA	pUTCCIISA Bc, pKTCIISA Bc
ASP/CIISABcNOSTOP/EcoRI	TCATCATCA**GAATTC**GATTTACTAAAATAACTATGA	pUTCIISA Bc, pKNTCIISA Bc
SP/IISBBc/BamHI	TCATCATCA**GGATCC**CGTGATTGTAGTGGTAAAAGA	pUTIISB Bc, pUTCIISB Bc
ASP/IISBBcNOSTOP/EcoRI	TCATCATCA**GAATTC**GATGATTTTCTTTTTAATTCTT	pUTIISB Bc
ASP/IISBBcSTOP/EcoRI	TCATCATCA**GAATTC**GAGCAAATGTAGAAAGAGTGTA	pUTCIISB Bc
SP/IISCBc/BamHI	TCATCATCA**GGATCC**CATGGCTGAAGTCAATGTGCA	All four BACTH vectors carrying SpoIISC Bc
ASP/IISCBcNOSTOP/EcoRI	TCATCATCA**GAATTC**GATGCATTTTCTTTTGTTCTTT	pUTIISC Bc, pKNTIISC Bc
ASP/IISCBcSTOP/EcoRI	TCATCATCA**GAATTC**GACTATGCATTTTCTTTTGTTC	pUTCIISC Bc, pKTIISC Bc
SP/CIISABc/BamHI2	TCATCATCA**GGATCC**GATTTCAGAAATATGGGG	pETDuetCIISA Bc
ASP/CIISABc/EcoRI	TCATCATCA**GAATTC**GATTCTGTCCTTATTTACTAA	
SP/IISBBc/KpnI	TCATCATCA**GGTACC**GTGATTGTAGTGGTA	pETDuetIISB Bc, pETDuetCIISAB Bc (via pETDuetCIISA Bc)
ASP/IISBBc/XhoI	TCATCATCA**CTCGAG**TGATTTTCTTTTTAA	
SP/IISABs/EcoRI	TCATCATCA**GAATTC**ATGGTTTTATTCTTTCAGATCATGGTCTGG	pBADIISA Bs, pBADIISAB Bs
ASP/IISABs/NcoI	TCATCATCA**CCATGG**TTCCATTATCCTTCACCTTC	pBADIISA Bs
ASP/IISBBs/NcoI	TCATCATCA**CCATGG**TTTAGTGTGATCATGCTTTT	pBADIISAB Bs
SP/IISCBs/PstI	TCATCATCA**CTGCAG**AGAGGATAATGTCAGGTGAT	pBADIISAC Bs
ASP/IISCBs/HindIII	TCATCATCA**AAGCTT**CAAAGACCATAAAAATCCCGGAGCCGCTCC	
SP/CIISABs/BamHI	TCATCATCA**GGATCC**CAAAAAACTGGCCGGCAGCGAGCTTGAAACA	All four BACTH vectors carrying CIISA Bs
ASP/CIISABsSTOP/EcoRI	TCATCATCA**GAATTC**TTATCCTTCACCTTCCTCCT	pUTCCIISABs, pKTCIISABs
ASP/CIISABsNOSTOP/EcoRI	TCATCATCA**GAATTC**GATCCTTCACCTTCCTCCTCAA	pUTCIISABs, pKNTCIISABs
SP/IISBBs/BamHI	TCATCATCA**GGATCC**CATGGAACGTGCGTTTCAAAACAGATGCGAG	pUTIISB Bs, pUTCIISB Bs
ASP/IISBBsNOSTOP/EcoRI	TCATCATCA**GAATTC**GATCCTTCACCTTCCTCCTCAA	pUTIISB Bs
ASP/IISBBsSTOP/EcoRI	TCATCATCA**GAATTC**TCATGCTTTTTTTCGTTTAT	pUTCIISB Bs
SP/IISCBs/BamHI	TCATCATCA**GGATCC**CGTGACATATAATAAATACAA	All four BACTH vectors carrying SpoIISC Bs
ASP/IISCBsNOSTOP/EcoRI	TCATCATCA**GAATTC**GATGCTTTTTTTCGTTTATACT	pUTIISC Bs, pKNTIISC Bs
ASP/IISCBsSTOP/EcoRI	TCATCATCA**GAATTC**GATTATTTTTTCTTCTTCAACT	pUTCIISC Bs, pKTIISC Bs

### Bacterial two-hybrid system

Fragments T25 and T18 from the adenylate cyclase bacterial two-hybrid system (Karimova et al., 1998) were fused with the C-terminal domain of SpoIISA, full-length SpoIISB and SpoIISC, all from both *B. cereus* and *B. subtilis*. Chromosomal DNA from *B. subtilis* PY79 and *B. cereus* ATCC 14579 were used as PCR templates. *E. coli* BTH101 was used as a host for testing protein-protein interactions. Cells were co-transformed with the relevant plasmid combinations and plated onto LB plates supplemented with 100 μg ml^−1^ ampicillin, 30 μg ml^−1^ kanamycin, 40 μg ml^−1^ X-Gal and 0.1 mM IPTG and grown for 48 h at room temperature.

### SDS-PAGE analysis

One dimensional SDS-PAGE was performed according to Laemmli (1970). Samples of whole cell lysates of recombinant-protein expressing *E. coli* BL21 (λDE3) cells, protein complexes, or purified protein samples were resuspended in sample buffer [4% SDS (w/v); 10% β-mercaptoethanol (v/v); 20% glycerol (v/v); 0.25 M Tris-Cl, pH 8] and boiled for 10 min. Denatured proteins were separated in 12% polyacrylamide gels. Due to the low molecular weight of *B. cereus* SpoIISC (6.6 kDa), this protein was analyzed using 16.5% Tricine–SDS-PAGE (Schägger and von Jagow, 1987), which better resolves such small proteins. As for the SDS-PAGE, samples of whole cell lysates of *E. coli* BL21 (λDE3) cells expressing recombinant SpoIISC and purified protein samples were resuspended in Novex sample buffer (Invitrogen, USA), then heated for 5 min in a boiling water bath and briefly spun down. The gels were run at 25 mA and stained with Coomassie brilliant blue R-250.

### Pull-down assay

Pull-down assays were used to confirm *in vitro* the interactions between *B. cereus* C-SpoIISA and SpoIISB, SpoIISC and *B. subtilis* C-SpoIISA. In order to investigate the interaction of His_6_-tagged *B. cereus* C-SpoIISA with S-tagged SpoIISB, the following proteins were isolated: His_6_-tagged C-SpoIISA, S-tagged SpoIISB and His_6_-tagged C-SpoIISA expressed together with S-tagged SpoIISB. *E. coli* BL21 (λDE3) competent cells were transformed with the pETDuetCIISA Bc and pETDuetIISB Bc plasmids (Table 1) for the overexpression of His_6_-tagged C-SpoIISA and S-tagged SpoIISB, respectively. Transformation with pETDuetCIISAB Bc was performed to obtain co-expression of His_6_-tagged C-SpoIISA with S-tagged SpoIISB. The resulting cell cultures were grown at 28°C in LB medium supplemented with 100 μg ml^−1^ ampicillin and 0.5% glucose. Recombinant protein expression was induced by the addition of IPTG to a final concentration of 0.5 mM, when the culture reached an OD_600_ of ~0.6. Cells were harvested 5 h after induction, centrifuged, and resuspended in solubilization buffer [20 mM Tris-Cl, pH 8; 150 mM NaCl; 10% glycerol (v/v); 10 mM MgCl_2_; 1 mM AEBSF]. Proteins were solubilized by overnight incubation at 14°C in the presence of 10 mM CHAPS (Sigma Aldrich). Samples were centrifuged for 30 min at 60 000 × g and 4°C. Soluble fractions were loaded onto a Ni Sepharose HP column (Amersham Bioscience) and washed; bound proteins were eluted with an imidazole step gradient from 0.2 M, to 0.4 M, 0.6 M and 1 M. The most concentrated fraction of the His_6_-tagged C-SpoIISA, that with 1M imidazole, was used in further experiments. The S-tagged *B. cereus* SpoIISB 0.2 M imidazole fraction was used as a control for non-specific binding to the Ni column. Finally, the 0.4 M imidazole fraction of SpoIISB was used in the assay to confirm that His_6_-tagged C-SpoIISA interacts with S-tagged SpoIISB. These proteins and the C-SpoIISA–SpoIISB protein complex were fractionated by 16.5% Tricine–SDS–PAGE. The fractioned proteins were transferred onto a nitrocellulose membrane and subsequently Western blotted.

The pull-down assay of His_6_-tagged *B. cereus* C-SpoIISA with untagged SpoIISC was performed similarly as described above. In this case, *E. coli* BL21 (λDE3) cells were transformed with pETDuet-Bc-HCIISAC for the interaction study and pETDuet-Bc-IISC (Table 1) to control for the non-specific binding of *B. cereus* SpoIISC to the Ni column.

### Glutaraldehyde crosslinking

The oligomeric state of recombinant *B. cereus* His_6_-C-SpoIISA was assessed by glutaraldehyde crosslinking. *E. coli* BL21 (λDE3) competent cells were transformed with pETDuetCIISA Bc, and protein expression was induced with 0.5 mM IPTG for 5 h at 28°C. Cells were then harvested and resuspended in a buffer containing 20 mM HEPES pH 7.5 and 150 mM NaCl and sonicated. The soluble fractions were centrifuged for 30 min at 60 000 × g and 4°C and then loaded onto a Ni Sepharose HP column (Amersham Bioscience). Proteins were eluted with an imidizole step gradient from 0.1 M to 0.2 M, 0.3 M and 1 M. For the crosslinking, 80 μg of protein was mixed with 5 μl of a freshly prepared solution of 2.3% glutaraldehyde to make a total volume of 100 μl. This reaction mixture was incubated for 30 min at 37°C and the reaction was then stopped by the addition of 10 μl of 1 M Tris-HCl, pH 8.0. The crosslinked molecules of *B. cereus* C-SpoIISA were loaded onto a 12% SDS-PAGE gel and detected by Western blotting.

### Western blotting

To visualize the interaction of *B. cereus* C-SpoIISA with the heterologous *B. subtilis* C-SpoIISA as well as the interaction of *B. cereus* C-SpoIISA with SpoIISB, we performed Western blotting using the general protocol of Ausubel et al. (1987). Briefly, proteins were fractionated by either 12% SDS-PAGE or 16.5% Tricine-SDS-PAGE and transferred onto a nitrocellulose membrane (Hybond ECL; Amersham Bioscience). To prevent non-specific binding, the membrane was treated using 5% non-fat milk in Tris-buffered saline with 0.05% Tween 20 (v/v). His_6_-tagged *B. cereus* C-SpoIISA was probed with an anti His_6_-tag monoclonal antibody (Novagen; catalog no. 70796-3) while S-tagged *B. subtilis* C-SpoIISA and S-tagged *B. cereus* SpoIISB were probed with an anti S-tag monoclonal antibody (Novagen; catalog no. 71549-3). Protein interactions were detected using antimouse horseradish peroxidase-conjugated secondary antibodies (Promega; catalog no. W402B).

### Gel filtration

To analyze the oligomerization of *B. cereus* C-SpoIISA using gel filtration, we developed a procedure for purifying untagged *B. cereus* C-SpoIISA. First, *E. coli* BL21 (λDE3) cells were transformed with the plasmid pET15b-Bc-CIISA. Next, the cell culture was grown at 28°C in LB medium supplemented with 100 μg ml^−1^ ampicillin. When the culture reached an OD_600_ of 0.6, the expression of untagged C-SpoIISA was induced with 0.5 mM IPTG. The cells were harvested 5 h after induction, centrifuged and resuspended in a resuspension buffer containing 50 mM glycine, pH 10; 50 mM NaCl; 10 mM MgCl_2_; 10% glycerol (v/v); and 1 mM AEBSF. The protein was solubilized by incubating at 14°C overnight in the presence of 10 mM CHAPS (Sigma Aldrich). The soluble fractions were cleared by centrifugation for 30 min at 60 000 × g and 4°C and loaded onto a HiPrep DEAE Sepharose FF 16/10 column (GE Healthcare Life Sciences), which had previously been equilibrated with a resuspension buffer containing 10 mM CHAPS. The protein eluted in the flow-through fraction and was loaded onto a HiPrep Q Sepharose HP 16/10 column (GE Healthcare Life Sciences), previously equilibrated with the same solution. The protein was eluted from this column with a continuous salt gradient ranging from 0.2 to 1 M NaCl. The purified protein was applied to a Superose 6 10/300 GL column (GE Healthcare Life Sciences) connected to an FPLC (GE Healthcare Life Sciences) instrument controlled by UNICORN 5.11 software, at a flow rate of 0.4 ml min^−1^. The elution was followed using UV absorbance at 280 nm.

### Dynamic light scattering measurements

DLS experiments were performed at 20°C on a Zetasizer Nano ZS instrument (Malvern Instrument) controlled by DTS software (version 5.1, Malvern Instruments Ltd). The instrument has a 90° scattering angle. The purified protein, at a concentration of 100 μM in a resuspension buffer at pH 8 containing 10 mM CHAPS, was filtered through 20 nm filters into a 40 μl cuvette. A single measurement consisted of 20 runs of 12 s each. All measurements were done in triplicate. The samples gave a clear signal (the *y*-intercept was 0.95) and required only moderate attenuation (set at 7).

### Bioinformatics analysis

Promoter analysis was performed using BPROM (Solovyev and Salamov, 2011). Identification of Rho-independent bacterial terminators was done using was done using ARNold web tool (Naville et al., 2011; http://rna.igmors.u-psud.fr/toolbox/arnold/). *B. cereus* SpoIISA membrane topology prediction was done using the MEMSAT3 and MEMSAT-SVM algorithms (http://bioinf.cs.ucl.ac.uk/psipred/; Nugent and Jones, 2009).

## Results and discussion

### The SpoIISABC toxin-antitoxin system

The SpoIIS toxin-antitoxin system in *Bacillus subtilis* consists of a SpoIISA toxin that is neutralized by a SpoIISB antitoxin (Adler et al., 2001; Florek et al., 2008). However, profiling of the condition-dependent transcription of *B. subtilis* revealed the presence of a third transcriptionally active region, denoted as S458 (Nicolas et al., 2012), located 55 bp downstream of *spoIISB* in the *spoIIS* operon, which we named *spoIISC*. Adler et al. (2001) identified two promoters in the *B. subtilis spoIIS* operon. The first promoter (P_A_) is located upstream of *spoIISA* and is important for regulating the expression of both *spoIISA* and *spoIISB*. The second promoter (P_B_) is located within the *spoIISA* gene and serves to regulate the expression of *spoIISB*. A promoter search using BPROM (see Materials and Methods) revealed a possible additional promoter (P_*C*_) downstream of *spoIISB* which could be used to regulate the expression of *spoIISC*. Its -35 sequence is 5′-TTCCTT-3′ and its -10 sequence is 5′-ACATATAAT-3′. In addition, a search for Rho-independent bacterial terminators using the ARNold tool identified the terminator (5′- GAAAAAATAAATCCCGGAG**CGG**CTCCGGGATTTTTATGGTCT -3′; letters in bold indicates bases contributing to the loop structure, underlined letters are bases forming the stem of terminator hairpin) immediately after the *spoIISC* STOP codon.

**Figure 1:**
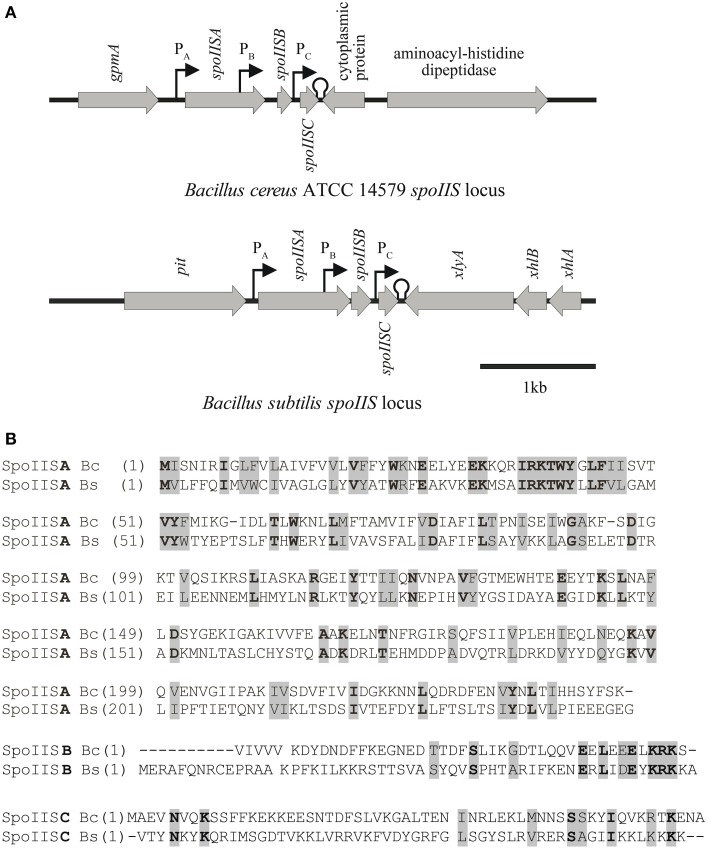
**Comparison of the ***spoIIS*** loci of ***Bacillus cereus*** and ***Bacillus subtilis***. (A)** Genomic organization of the *spoIIS* locus in *B. cereus and B. subtilis*. **(B)** Alignment of the SpoIIS proteins of *B. cereus* (Bc) and *B. subtilis* (Bs). Amino acids printed in normal weight on a gray background indicate similar amino acids, while bold weight on a gray background indicates identical amino acids.

We previously found that a two-component SpoIIS system also exists in *B. cereus* (Florek et al., 2008). The position of its locus on the chromosome is completely different from that of the *spoIIS* operon in *B. subtilis*. While the *B. subtilis spoIIS* operon is 115° away from the origin of replication, the *B. cereus spoIIS* locus is 158° away. The *B. cereus spoIIS* operon consists of *spoIISA* (BC_2436), which encodes a 245-residue SpoIISA-like protein, and BC_2437, which encodes a hypothetical protein with 58 residues. As shown in Florek et al. (2008), BC_2437 is found 316 bp downstream of the *spoIISA*-like gene and was named *spoIISB* since its SpoIISB-like product was able to neutralize the toxicity of the SpoIISA-like protein in *E. coli*. Prompted by the identification of a putative third transcript in the *B. subtilis spoIIS* operon (Nicolas et al., 2012), we revisited the bioinformatics analysis of the *B. cereus spoIIS* operon and found that the *B. cereus spoIIS* operon also likely contains three genes: the BC_2436 ORF encoding a 245-residue SpoIISA-like protein; a 138-bp ORF 103 bp downstream of this gene, which encodes a 45-residue, putative SpoIISB; and a further 72 bp downstream of that, the BC_2437 ORF, which encodes the 58-residue protein we had previously called SpoIISB, but which we now call SpoIISC (Figure 1; Florek et al., 2008). As in the *B. subtilis* analysis, BPROM identified putative promoters in this operon. *B. cereus spoIISA* appears to be driven by the putative promoter P_A_, the putative P_*B*_ promoter for controlling *spoIISB* gene expression is found within the *spoIISA* gene, and the putative P_C_ promoter that likely regulates the expression of *spoIISC* is located downstream of the *spoIISB* gene. ARNold tool predicts that a Rho-independent bacterial transcription terminator, with the sequence 5′-AA AGAACAAAAGAAAATGC**ATAGA**GCATTTTCTTTTGTTTTTTT A-3′ (letters in bold indicates bases contributing to the loop structure, underlined letters are bases forming the stem of terminator hairpin). This sequence overlaps with the end of *B. cereus spoIISC* gene (Figure 1A).

The presence of three promoters in the *spoIIS* locus may be due to the different conditions under which the expression of individual genes is induced. The transcription of all three *B. subtilis spoIIS* genes is clearly induced during sporulation, but during nutrient deprivation only the *spoIISA* and *spoIISB* genes are transcribed (Nicolas et al., 2012). Moreover, there are conditions which induce transcription of only one of these genes: *spoIISA* is transcribed during ethanol stress, *spoIISB* during swarming and at high cell density, and *spoIISC* during biofilm formation (Nicolas et al., 2012).

Both *B. subtilis* and *B. cereus* SpoIISA-SpoIISB systems are clear examples of type II TA systems (Adler et al., 2001; Florek et al., 2011). The *spoIIS* operon has been identified only in Bacilli, and only a low level of homology can be detected between the SpoIIS proteins of *B. subtilis* and *B. cereus* (Florek et al., 2008). SpoIISA proteins display 17.3% identity and 30.2% similarity, while the SpoIISB proteins have only 12.5% identity and 17.9% similarity. The SpoIISC proteins have the lowest homology, with only 8.6% identity and 15.5% similarity. On the other hand, the SpoIISB and SpoIISC proteins from one of these organisms exhibit a higher level of homology with each other than with their counterparts in the other organism. Thus the *B. subtilis* SpoIISB and SpoIISC proteins show 37.5% homology and 12.5% identity while *B. cereus* SpoIISB and SpoIISC have 35.6% similarity and 27.1% identity (Figure 1B).

Bacterial type II TA systems are normally organized so that the first gene in the operon codes for the antitoxin and the toxin is positioned farther downstream; both genes are usually preceded by their own promoters. This arrangement ensures an abundance of antitoxin is produced to prevent toxin activity when it is undesirable. One exception to this arrangement is the *higBA* TA module in pathogenic *Proteus* species (Hurley and Woychik, 2009). As noted above, the *spoIIS* system is another, with the toxin preceding its two putative antitoxin genes. The SpoIIS TA system is unusual in another way as well. The typical type II TA system is a two-component system, but the SpoIIS TA system consists of three components: the SpoIISA toxin, the SpoIISB antitoxin and the third component SpoIISC (a likely antitoxin). Other three-component type II TA systems have previously been reported, including the ω**-ε**-ζ TA module encoded by the *Streptococcus pyogenes* plasmid pSM19035, the *paaR-paaA-parE* TA module encoded by *E. coli* O157:H7, and the *pasA*/*pasB*/*pasC* module of the *Thiobacillus ferrooxidans* plasmid pTF-FC2 (reviewed in Unterholzner et al., 2013). In all of these systems, at least one of the three components is involved in autoregulating the operon. There is presently no information about whether the expression of the *spoIIS* operon in *Bacilli* is autoregulated.

### The *spoIISB* and *spoIISC* genes encode antitoxins in *Bacillus cereus*

*B. subtilis* transcription analysis by Nicolas et al. (2012) and in this study have revealed that the *spoIIS* operon is formed by the *spoIISA, spoIISB*, and *spoIISC* genes. In *E. coli, B. subtilis* SpoIISA inhibited bacterial growth and SpoIISB was able to neutralize SpoIISA toxicity (Florek et al., 2008). Previously, we observed that *B. cereus* SpoIISA, like *B. subtilis* SpoIISA, has a toxic effect on *E. coli* growth (Florek et al., 2008), but at that time, we had incorrectly designated ORF BC_2437 as *spoIISB*. A new bioinformatics analysis, prompted by the likely existence of a third gene in the *B. subtilis spoIIS* operon by Nicolas et al. (2012), shows that BC_2437 indeed contains *spoIISC* and that SpoIISB is a 45-residue protein of unknown function encoded by a small ORF (only 138 bp) located between the *spoIISA* and *spoIISC* genes.

**Figure 2:**
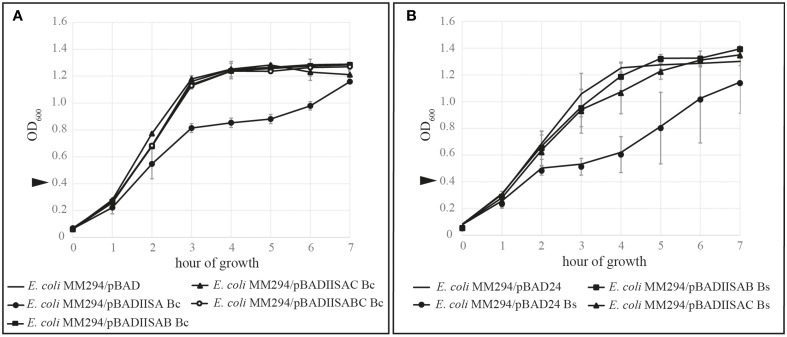
**Kill/rescue assay in ***E. coli*** MM294**. In order to test the ability of SpoIISC to act as an antitoxin for SpoIISA, SpoIIS proteins were expressed alone or in combination in *E. coli* cells. All results are mean values of three independent replicates and the bars represent 1 SD. The growth of *E. coli* cells expressing the SpoIISA toxin (circle) was inhibited while those cells expressing either the SpoIISAB complex (square) or the SpoIISAC complex (triangle) had wild-type growth (no marker). Arrows indicate the addition of 0.02% arabinose to induce expression. **(A)** The effect of the *B. cereus* SpoIIS proteins on the growth of *E. coli* MM294 cells. **(B)** The effect of the *B. subtilis* SpoIIS proteins on the growth of *E. coli* MM294 cells.

To determine if *B. cereus* SpoIISB and SpoIISC are both able to neutralize the toxicity of *B. cereus* SpoIISA in *E. coli*, the corresponding genes *spoIISAB* and *spoIISAC* were cloned into pBAD24 vectors under the control of arabinose-inducible P_BAD_ promoters to generate pBADIISAB Bc and pBADIISAC Bc. These plasmids were subsequently introduced into *E. coli* MM294 cells. The growth of these transformed cells, together with the control strains IB890 (*E. coli* MM294 / pBAD24) and IB926 (*E. coli* MM294/pBAD-BCIISA) (Florek et al., 2008), was monitored after the induction of protein expression. As found previously (Florek et al., 2008), the growth of *E. coli* cells expressing only *B. cereus* SpoIISA was inhibited. On the other hand, both SpoIISB and SpoIISC were able to neutralize the toxicity of SpoIISA: the growth curves of those strains which expressed both SpoIISA and either the SpoIISB antitoxin or SpoIISC were similar to that of the wild-type IB890 *E. coli* cells (Figure 2A). Because *B. cereus* SpoIISB and SpoIISC disturb SpoIISA toxicity when expressed in *E. coli* cells, it can be concluded that both *spoIISB* and *spoIISC* encode antitoxins and that they are likely to have similar functions as the antitoxins in *B. subtilis*. Indeed, an identical set of experiments using the *B. subtilis* genes rather than the *B. cereus* ones gives very similar results (Figure 2B).

### The interactions of SpoIIS proteins in a bacterial two hybrid system

**Figure 3:**
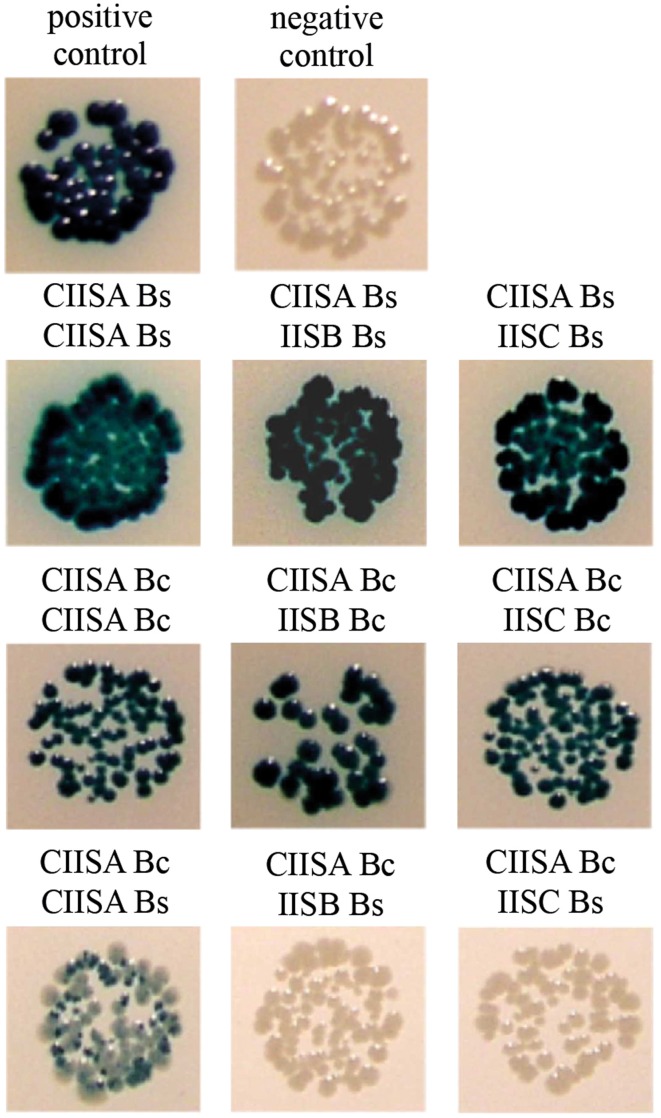
**Interaction study of the SpoIIS proteins using the BACTH system**. Since fusions with SpoIIS proteins in both orientations were positive in some cases, only representative ones were selected. A strain expressing a pair of leucine zipper proteins, T25-Zip and T18-Zip, served as the positive control; the negative control was a strain expressing the pair T25-CIISA Bc and T18-Zip. Abbreviations: Bc, *B. cereus*; Bs, *B. subtilis*; CIISA, C-terminal domain of SpoIISA; IISB, SpoIISB; IISC, SpoIISC.

The clearest evidence that *B. subtilis* SpoIISA and SpoIISB directly interact can be found in the crystal structure of the C-terminal domain of SpoIISA in complex with SpoIISB (Florek et al., 2011). To analyze the protein–protein interactions of the *B. cereus* SpoIIS proteins *in vivo*, we made use of the bacterial adenylate cyclase two hybrid system (Karimova et al., 1998). Like *B. subtilis* SpoIISA, *B. cereus* SpoIISA is predicted to be a membrane protein with three membrane-spanning segments. However, we decided to use only the cytoplasmic domains in this protein–protein interaction study, since the whole SpoIISA protein is toxic for *E. coli* as we have shown previously. We prepared fusions of the C-terminal domain of *B. cereus* SpoIISA, SpoIISB, and SpoIISC with the adenylate cyclase fragments T25 and T18. All possible interactions were tested and compared with those of similar SpoIIS fusion proteins from *B. subtilis* (Figure 3).

Our results confirmed the dimerization of *B. subtilis* C-SpoIISA as well as the interaction of *B. subtilis* C-SpoIISA with SpoIISB described in Florek et al. (2011). A positive interaction was also observed for *B. subtilis* C-SpoIISA with SpoIISC (Figure 3). Finally, we found that the *B. cereus* C-terminal domain of SpoIISA can interact with another C-SpoIISA protomer, with SpoIISB and with SpoIISC (Figure 3).

### *B. cereus* SpoIISB and SpoIISC are able to bind the C-terminal domain of SpoIISA *in vitro*

**Figure 4:**
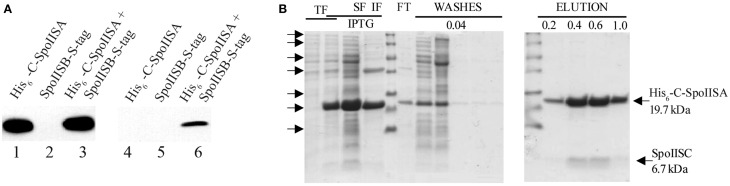
**Pull-down assays of ***B. cereus*** SpoIISB and SpoIISC with C-SpoIISA**. The soluble fractions of lysed bacterial cells were applied to a Ni Sepharose HP column. The eluted proteins were identified by Western blotting **(A)** and Coomassie brilliant blue R-250 staining **(B)**. **(A)** In the Western blot, the eluted proteins were probed with an anti-His_6_ monoclonal antibody (lanes 1–3) or with an anti-S monoclonal antibody (lanes 4–6). Lanes 1 and 4 contain purified His_6_-tagged C-SpoIISA, lanes 2 and 5, purified S-tagged SpoIISB. S-tagged SpoIISB does not bind a Ni Sepharose HP column. Lanes 3 and 6 show that His_6_-tagged C-SpoIISA can pull down S-tagged SpoIISB and therefore that there is an interaction between them. **(B)** A pull-down assay showing an interaction between His_6_-tagged C-SpoIISA and SpoIISC when both proteins are co-expressed. TF, total fraction; SF, soluble fraction; IF, insoluble fraction; FT, flow-through fraction, 0.04; 0.2; 0.4, 0.6, and 1.0—molarity of imidazole used in washing and elution. The arrows mark the following positions on the protein ladder from top to bottom: 116, 66.2, 45, 35, 25, 18.4, and 14.4 kDa.

To analyze these protein–protein interactions *in vitro*, we prepared three derivatives of the pETDuet recombinant expression plasmid, each containing one of the following genes, all under the control of an IPTG-inducible T7 promoter: a gene coding for a His_6_-tagged *B. cereus* C-SpoIISA, an S-tagged SpoIISB and an untagged SpoIISC (Table 1). We found that His_6_-tagged C-SpoIISA binds the Ni column and that S-tagged SpoIISB and untagged SpoIISC creates a tight complex with C-SpoIISA which can be eluted by a solubilization buffer step gradient containing 0.1–1 mM imidazole (Figure 4).

The interaction of *B. cereus* His_6_-tagged C-SpoIISA with S-tagged SpoIISB was confirmed in a pull-down assay by the co-elution of both proteins from a Ni column. When SpoIISB is co-expressed in *E. coli* together with His_6_-tagged C-SpoIISA, the His_6_-tagged C-SpoIISA binds the Ni column, and since S-tagged SpoIISB binds C-SpoIISA, the two are pulled down together as a complex during elution with 0.4 M imidazole (Figure 4A). This complex could be detected by Western blotting using an anti-His_6_-tag monoclonal antibody to identify His_6_-tagged C-SpoIISA (Figure 4A, lane 3) and an anti-S-tag monoclonal antibody to identify the S-tagged SpoIISB (Figure 4A, lane 6).

A similar approach was used to test the interaction of untagged *B. cereus* SpoIISC with His_6_-tagged C-SpoIISA *in vitro* (Figure 4B). *B. cereus* SpoIISC expressed in *E. coli* BL21 (DE3) appears in the insoluble fraction of the cell lysate according to 16.5% Tricine/SDS–PAGE (data not shown). However, when co-expressed with *B. cereus* His_6_-tagged C-SpoIISA in the same cells, they form a complex which is able to pull SpoIISC out of the insoluble fraction. The whole complex can then be solubilized and purified from the soluble fraction by affinity chromatography.

### *B. cereus* C-terminal domain of SpoIISA forms an oligomer

**Figure 5:**
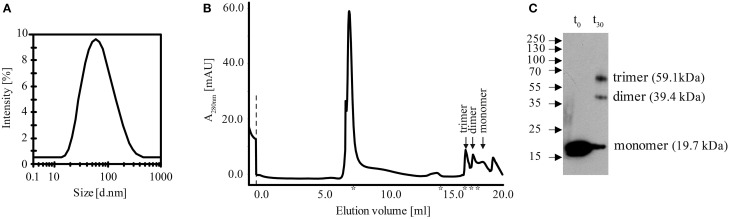
**Analysis of the multimeric state of ***B. cereus*** C-SpoIISA. (A)** Dynamic light scattering analysis of C-SpoIISA oligomer. Size distribution (by intensity) of *B. cereus* C-SpoIISA, at 20°C, average hydrodynamic radius = 55 nm. **(B)** Gel filtration of C-SpoIISA. The stars indicate the positions at which the following protein standards eluted from the column (left to right): 2000, 450, 66, 45, and 29 kDa. **(C)** Western blot analysis of glutaraldehyde-crosslinked His_6_-tagged C-SpoIISA.

The crystal structure of the *B. subtilis* SpoIISA C-terminal domain shows that the protein dimerizes by forming a four-helix bundle using the first and last α-helices of each molecule (Florek et al., 2011). Our bacterial two-hybrid experiments showed that *B. cereus* C-SpoIISA interacts with other *B. cereus* C-SpoIISA molecules (Figure 3), suggesting that this molecule also forms oligomers. The oligomeric form of C-SpoIISA was examined by measuring the hydrodynamic radius of dissolved particles using dynamic light scattering. A cumulant analysis showed that the sample was monomodal (i.e., had only one peak, Figure 5A), and was polydisperse, with a polydispersity index of 0.255 and an overall polydispersity of 50.32%. The polydispersity indicates broader particle size distribution, and thus the hydrodynamic radius and corresponding molecular mass cannot be reliably calculated.

The SpoIISA oligomerisation was examined further by size-exclusion chromatography of C-SpoIISA using a Superose 6 10/300 GL column. In this analysis, most of the protein appeared in the void volume fraction of the column, which was determined from the elution of Blue dextran 2000 (~2000 kDa, Pharmacia) (Figure 5B). Three small peaks were detected, however, and likely correspond to the 59.1 kDa trimer, the 39.4 kDa dimer and the 19.7 kDa monomer of C-SpoIISA. The existence of monomeric, dimeric and trimeric states was confirmed by glutaraldehyde crosslinking (Figure 5C), but the existence of higher oligomeric forms could not be confirmed because such large species would not have been able to enter the crosslinking gel.

Taken together, the above results indicate that *B. cereus* C-SpoIISA is able to form higher multimers, even if their nature is unclear. In this respect, its behavior differs from that of *B. subtilis* C-SpoIISA, which formed only dimers (Florek et al., 2011). Whole *B. subtilis* SpoIISA does seem to form higher oligomers, but this seems to require its N-terminal transmembrane domain rather than just its C-terminal cytosolic domain (Makroczyová et al., 2014). Earlier studies also suggested that whole SpoIISA oligomerizes, and moreover suggested that it forms holin-like pores (Adler et al., 2001). Whether either the *B. subtilis* or *B. cereus* proteins actually do form such pores remains unknown, however.

Finally, this study describes the SpoIISC protein, a third component of the *spoIIS* locus. This protein serves as an antitoxin and shows similarity to SpoIISB. The presence of two antitoxin genes in the *spoIIS* locus of both *B. subtilis* and *B. cereus* naturally poses the question of the role of such duplication. One possibility is that the different proteins are linked to different conditions under which they might be expressed, as was shown for *B. subtilis* SpoIIS system (Nicolas et al., 2012). They may also act as transcription regulators, as some other antitoxins are known to. It is also possible that their different amino-acid compositions could affect their affinity for SpoIISA, leading to different degrees of inhibition. In any case, our results show that the SpoIIS TA system is much more complex than had previously been thought.

### Conflict of interest statement

The authors declare that the research was conducted in the absence of any commercial or financial relationships that could be construed as a potential conflict of interest.
